# Probiotic mixture improves fatty liver disease by virtue of its action on lipid profiles, leptin, and inflammatory biomarkers

**DOI:** 10.1186/s12906-016-1540-z

**Published:** 2017-01-13

**Authors:** Hessah Mohammed Al-muzafar, Kamal Adel Amin

**Affiliations:** Department of Chemistry, Faculty of Science, University of Imam Abdulrahman Alfaisal (Dammam), P.O. 383, Dammam, 31113 Saudi Arabia

**Keywords:** Probiotics, HFSD, Lipid profile, Leptin, Inflammation, NAFLD biomarkers

## Abstract

**Background:**

A high fat diet has an essential role in the pathogenesis of non-alcoholic fatty liver disease (NAFLD). This condition is characterized by hepatic fat accumulation (steatosis) and is associated with obesity, diabetes, and fibrosis or cirrhosis of the liver. Probiotics may be useful in the treatment of steatosis. This study examined the effects of an ingested probiotic formulation on the lipid profiles, liver functions, leptin levels, and inflammatory marker levels of rats with NAFLD that had been induced via high fat and sucrose diet (HFSD).

**Methods:**

Young male albino rats were randomly divided into three groups: a control group that was fed a standard diet; a second group that was fed a HFSD; and a third group that was given both a HFSD and ingestible probiotic mixtures. The groups were fed these diets for 16 weeks, and were then examined.

**Results:**

HFSD-only rats showed hypertriglyceridemia, hypercholesterolemia, and elevated low density lipoprotein (LDL) levels, and their serum alanine transaminase (ALT) and bilirubin levels were significantly higher than those of the control group. Compared to rats on the standard diet, HFSD-only rats showed higher levels of tumor necrosis factor alpha (TNF-α) and interleukin 6 (IL-6), increased serum leptin levels, and increased resistin hormone levels in the adipose tissues. In the third group, the inclusion of the probiotic mixture seemed to ameliorate the effects of the HFSD diet. The NAFD + probiotics group showed improved lipid profiles, better leptin and resistin levels, and better TNF-α and IL-6 levels than the NAFD-only group. They also showed no signs of NAFLD.

**Conclusions:**

The probiotic mixture showed promise as a treatment for NAFLD pathogenesis, and may improve HFSD-induced steatosis through its effects on leptin, resistin, inflammatory biomarkers, and hepatic function markers. We also established that gut microbiota-mediated regulation of lipid profiles was dependent on dietary lipids and carbohydrates.

## Background

The prevalence of non-alcoholic fatty liver disease (NAFLD) has increased in several societies, and its treatment and management have become economic and public health priorities [1, 2]. A clear understanding of NAFLD, and other manifestations of metabolic syndrome (MetS), is required to develop effective therapies and improve existing ones [3]. Prevention and treatment of NAFLD are expensive and lengthy processes; further efforts are needed to help people to avoid unhealthy diets, maintain healthy lifestyles, and implement existing therapies using antioxidants, oral hypoglycemics, and fat-lowering agents [4].

The intestinal epithelium is a mucosal barrier that is part of our innate immune system, and it can release proinflammatory cytokines such as interleukin 6 (IL-6) and TNF-α In response to enteric pathogens [5]. The non pathogenic bacteria that comprise the intestinal microflora perform several functions that assist the host organism, including the establishment of a protective barrier over the intestinal epithelium that serves the host both metabolically and trophically. Therefore, any abnormality or disruption in the microbiota (dysbiosis) may have deleterious consequences on the general health of the host [6].

The digestive tract harbors a microbiota that plays vital roles in the onset and progress of health and disease [7, 8]. The internal environment of microbiota is not inert, and can be influenced by dietary components specially fat. Feeding an animal a high fat sucrose diet (HFSD) resulted in obesity; this is a disease model that we developed in previous work [9] and which is used to imitate the obese phenotypes observed in the human populations of Western and more developed societies. HFD causes intestinal bacterial overgrowth and leads to dysbiosis, which promotes endogenous signals that have pathogenic roles in hepatic insulin resistance and hepatic fat accumulation [10]. Previous studies have shown that while a sucrose-rich diet does not necessarily result in obesity, it can induce other issues: e.g., hypertrophy in adipocytes, glucose intolerance, hyperinsulinemia, hyperlipidemia, and liver steatosis (a condition in which the abnormal deposition of lipids inside a cell occurs) [11]. Thus, sucrose-rich diets can cause health problems and have far-reaching, deleterious effects [12].

Gut bacterial metabolites with short chain fatty acids (SCFAs), can affect host metabolism. Hippurate is considered a risk marker for diabetes, cardiovascular diseases, and obesity, and acts as a signal for lipolysis and food intake. Gut bacterial cellular components, such as lipopolysaccharides (LPSs), flagella, structural lipids, and peptidoglycans, can affect the host’s immune response, induce insulin resistance, and cause MetS [13, 14].

Probiotics are live microorganisms that provide health benefits to the host when ingested in appropriate amounts [15]. A goal of probiotic use is the creation of symbiotic relationships between the human host and naturally occurring microorganisms, generating positive effects on the human host’s overall health and ability to resist illness [16]. Probiotics are safe and widely accepted by the public. Over the past five years, they have developed into what is perceived as a natural treatment against harmful factors associated with MetS and related disorders [17–19]. Certain probiotics (*Bifidobacterium* or *Lactobacillus spp*, *Streptococcus thermophiles*, and *Akkermansia muciniphila*) have been used to decrease obesity and type 2 diabetes in HFD-fed model animals [20, 21].

Probiotics may attenuate MetS by modulating gut microbiota, and have the capacity to modulate metabolic phenotypes [19, 22, 23]. Consequently, there can be different probiotic effects on the gut microbiota composition, which can lead to distinct consequences and improvements on the host’s metabolic health. However, these processes are still poorly understood; this is probably due to insufficient steatosis models, insufficient data, and the existence of contradictory data, that may be resulted from low dose, short duration of administration. Some probiotics, including *Bifidobacterium* and *Lactobacillus*, affect the cycle of the intestinal mucosa barrier. Furthermore, certain probiotics types such as *L. acidophilus* and *L. rhamnosus* can protect the mucosal immune system by preventing the adhesion of harmful bacteria to the gut endothelial lining, and by reducing elevated levels of fecal TNF-α [24].

The goal of this study was to investigate the effects of probiotic ingestion on the lipid profiles and hepatic functions of host animals, because of its accepting natural therapies rather than taking pharmaceuticals and to answer certain questions relating to the effects of gut bacteria. For example, does the gut probiotics play a role in NAFLD pathogenesis? What is the mechanism by which the gut probiotics helps the host animal to maintain homeostasis? The effects of probiotic ingestion on NAFLD and other metabolic disorders have not yet been demonstrated, and further research is needed to determine optimal probiotic strains, algorithms of administration, and subsequent effects on organ functions. Probiotics appear to have great potential in terms of benefiting human health, and this justifies the undertaking of additional research [25].

Some lactic acid bacteria (LAB) have been shown to reside in the gastrointestinal tract (GIT) [24], with beneficial functions that include cytokine regulation, maintenance of intestinal permeability, and control of the immune response [26, 27]. However, the mechanisms driving the immunological effects are not fully understood. Based on previous work, we suggest that there is poor communication between LAB, probiotics, and immune cells; we also speculate that pro- and anti-inflammatory mediators are regulated by probiotics and have potential roles in the immune response [26, 27]. Bacterial surface proteins, metabolites, and secreted proteins can stimulate cytokine secretion and can activate NF-κB in cells in vitro [28]. However, the effects of these bacterial products on host cytokine secretion and its blood level require in vivo support and exploration to be better understood; our study employs a mixture of bacteria at various concentrations, and laboratory rats as the experimental in vivo components, to address this.

Our aim is to induce NAFLD disease in rats, using a HFSD, to produce an animal model of human steatosis in which we may monitor liver function biochemical markers, lipid profiles, adiposity, and inflammation hormone levels. We explore the subjects’ microbiota as affected by the application of probiotics, and we propose possible mechanisms for steatosis treatment via the effects of probiotics on disease markers. More specifically, we focus on the use of a gut probiotics mixture and its effects on the specific biomarkers related to hepatic steatosis and its associated disorders.

## Methods

### Diet

Two types of diet were used: 1) a control rat chow and 2) a HFSD (purchased from PE Enterprise, grain Riyadh Branch, KSA, experimental animal feed #1005), . The control rat chow consisted of protein concentrates (350 g of soya bean, whey protein, and meat), corn (600 g), calcium carbonate, dicalcium phosphate, sodium chloride, magnesium oxide, and vitamins (50 g). This standard or normal rat diet consisted of 65% carbohydrates (60% starch + 5% sucrose), 5% fat, 20% crude protein, 5% vitamins and minerals, and 5% dietary fiber. The metabolic energy of this diet was 2813 kcal/kg, with 8% of this energy coming from fat. The HFSD was prepared from protein concentrates (400 g), corn (350 g), 100% pure vegetable ghee (200 g at 9 kcal/g), and vitamins and minerals (50 g), according to the formulations used by Amin et al. [9]. The HFSD consisted of 55% carbohydrates, 20% fat (200 g SFA/kg food), 20% crude protein, and 5% vitamins, minerals, and dietary fiber. The metabolic energy of this diet was 5100 kcal/kg, with 59% of this energy coming from fat. In addition, sucrose (1 g/50 mL water) with high fat was used for the induction of NAFLD in rats.

### Experimental animals

Sixty, 6 week-old male rats were supplied by King Abdulaziz City for Science and Technology (KACST), with an average weight of 80–90 g. All rats were kept under observation for one week upon arrival. All animals were housed individually in plastic cages at 24 ± 3 °C, in 12 h light/12 h dark cycles, with humidity 40–60%, in the laboratories of KACST, University of Dammam. Rats had free access to water and diet.

### Preparations for treatments

#### Probiotics

A commercially available probiotic described as a concentrated source of naturally occurring microorganisms (AVI-5-BAC packet; Sure Pharmaceutical USA, Lombard, IL, USA) was administered at a dose of 1 g per kg of food. This probiotic mixture contained *Lactobacillus acidophilus* (10 × 10^8^ CFU/g), *Lactobacillus plantarum* (9.8 × 10^7^ CFU/g), *Bifidobacterium bifidum* (2 × 10^6^ CFU/g), *Bacillus subtilis* fermentation extract (50 g per kg of product), *Aspergillus oryzae* fermentation extract (50 g per kg of product), and maltodextrin (added to 1 kg).

#### Chemicals

Sera were analyzed for total cholesterol (TC), triglycerides (TGs), and high density lipoprotein (HDL) levels, LDL, and bilirubin levels. These parameters determined colorimetrically using kits purchased from Human Gesellschaft fur Biochemica und Diagnostica mbH (Wiesbaden, Germany). Leptin and resistin were measured using an ELISA kit from SPI Bio-Product PTY Ltd (# A051760 and # A05179) (Montigny le Bretonneux France). IL-6 and TNF-α ELISA detection kits were purchased from Abcam (ab100712 and ab46105).

### Experimental design and animal grouping

The experiments lasted 16 weeks and were divided in two periods: 1) induction of steatosis (weeks 1–12), and 2) treatment (weeks 12–16). Sixty rats were used for this study, and were randomized into three groups, each group containing 20 rats. For the duration of the experiment, body weights were recorded weekly; mean body weights, and weight gains, were calculated.

#### Induction of hepatosteatosis

From weeks 1–12, the animals were divided into two groups. The negative control group (20 rats) was fed the standard rat chow diet for the duration of the experiment (16 weeks), and the HFSD group (40 rats) was maintained on the HFSD.

#### Probiotic treatment

From weeks 12–16 (the treatment phase), the HFSD group (*n* = 40) was divided into two groups. Group 1 (*n* = 20) continued on the HFSD (positive control group), and group 2 received concomitant supplementation of probiotics at doses of 1 g per 1 kg of HFSD (treatment group).

### Blood and tissue sampling

Blood samples were collected from the medial canthus of the eye, using a microhematocrit capillary tube, during the fasting period. To collect the sera, blood samples were collected in dry glass centrifuge tubes and were allowed to clot at 24 ± 3 °C before being centrifuged at 1400 *g* for 20 min. The clear, non-hemolysed supernatant sera were aspirated using clean, dry disposable plastic syringes, and were kept at −80 °C for subsequent biochemical measurements. Upon completion of the experiment, rats were sacrificed under anaesthesia, with blood and liver extracted for later analysis. Portions of the liver was fixed with 10% formalin for histopathological examination.

### Biochemical analyses of serum and histopathological examinations

Blood samples were used to carry out the biochemical analysis of the lipid profiles, including TG, TC, LDL, and HDL. We also tested for hepatic function by detecting levels of alanine aminotransferase (ALT), bilirubin, and albumin. Levels of the adipose tissue hormones leptin and resistin, and of the cytokines TNF-α and IL-6, were evaluated as inflammatory markers. Histopathological examinations were also conducted using hematoxylin and eosin (H&E) stain and examining tissues under the microscope to inspect hepatocyte structure and shape, the presence or absence of inflammatory cells, the presence or absence of fat globules between and within the hepatocytes, and any degenerative changes in hepatocytes. Degrees of NAFLD were evaluated at low magnification (4×, 10×) and higher magnification. Hepatocellular steatosis, fat globules, mild to moderate macro and microvesicular steatosis, inflammatory cells and lobular inflammation were scored and the severity was graded [29]. Based on the percentage of the total area affected, NAFLD categorized into score 0 (5%) considered normal, score 1 (5–33%) as mild, score 2 (33–66%) as moderate, and score 3 (66%) as severe NAFLD.

### Statistical analysis

Results are displayed as mean ± SEM, and were statistically analyzed using one-way analysis of variance (ANOVA), followed by the Tukey-Kramer method for post-hoc analysis. Values were considered significant when *p* < 0.05; the different superscript letters (a, b, c) indicate significant variations at *P* < 0.05 in the tables. Statistical analyses were performed using the GraphPad Prism 6 software application (San Diego, CA, USA).

## Results

At baseline, there was no significant difference in body mass between groups (*p* = 0.65). During the course of the experiment, final and mean body weight gains indicated significant increases in the HFSD group compared with the normal or negative control group. Probiotic treatment improved the final and mean body weight gain values within the HFSD group (Table 1).

**Table 1:** Effects of normal diet and HFSD on body weight in rats

	Normal	HFSD	HFSD + Probiotic
Initial body weight (g)	109.4 ± 4.52	101.6 ± 2.22	101.6 ± 2.22
Final Body weight gain	227.9 ± 15.36^a^	268.7 ± 8.450^b^	238.7 ± 9.43^a^
Mean body weight gain	60.03 ± 4.05^a^	71.78 ± 2.90^b^	64. 8 ± 3.80^a^

These values represent means and standard errors, the different superscript letters indicate significant variations at *P* > 0.05

The groups displayed differences in sera lipid profiles. The HFSD rats displayed significant increases in levels of TG, TC (*p* < 0.001), and serum LDL (*p* < 0.01), while also showing a significant decrease in levels of HDL (*p* < 0.01), compared to rats fed the normal diet (Table 2). The group that was fed the probiotic mixture showed significantly improved serum TG, TC, and LDL levels compared to the group that was fed the HFSD only (Table 2). There were also a significant increases in ALT activity, and in total and direct bilirubin, in the HFSD group compared to the normal group; this indicated the deleterious impact of the HFSD on hepatocytes. The group given the probiotic mixture displayed improved levels of these hepatic markers when compared to the HFSD-only group (Table 3). Furthermore, the HFSD-fed group showed significant increases in serum leptin and resistin hormone levels compared to the group fed the normal diet (Table 4); administering the probiotic mixture appeared to regulate these hormonal imbalances. Moreover, NAFLD in the HFSD-fed group resulted in significant increases in IL-6 and TNF-α levels compared to the groups fed the normal diet, while the group that was given the probiotic mixture appeared to recover from these changes.

**Table 2:** Effects of probiotics on lipid profiles in HFSD-fed rats

	Normal	HFSD	HFSD + probiotics
TG (mg/dL)	111.03 ± 9.01^a^	229.33 ± 7.62^b^	203.0 ± 5.8^c^
TC (mg/dL)	78.08 ± 4.35^a^	110.6 ± 2.19^b^	98.02 ± 2.5^c^
LDL (mg/dL)	35.7 ± 2.33^a^	64.5 ± 6.59^b^	38.0 ± 2.26^a^
HDL (mg/dL)	88.99 ± 8.082^a^	37.79 ± 4.52^b^	51.28 ± 7.03^b^

Values represented as means and standard errors, the different superscript letters describe a significant difference at *P* > 0.05

**Table 3:** Effects of probiotics on liver function in HFSD fed rats

	Normal	HFSD	HFSD + probiotics
ALT (U/L)	30.8 ± 1.94^a^	49.3 ± 4.39^b^	38.0 ± 2.79^a^
Albumin (g/dL)	3.13 ± 0.19^a^	3.00 ± 0.20^a^	3.08 ± 0.23^a^
T. proteins (g/dL)	8.41 ± 0.69	8.41 ± 0.56	7.88 ± 0.69
T. Bilirubin (mg/dL)	1.42 ± 0.16^a^	2.36 ± 0.23^b^	1.38 ± 0.18^a^
D. Bilirubin (mg/dL)	0.79 ± 0.09^a^	2.08 ± 0.1^b^	1.28 ± 0.16^c^

Values represented as means and standard errors, the different superscript letters mean a significant difference at *P* > 0.05

**Table 4:** Effects of probiotics on inflammatory markers and hormone levels in HFSD-fed rats

	Normal	HFSD	HFSD + probiotics
Leptin (pg/mL)	1525.2 ± 166.41^a^	2525.29 ± 153.7^b^	2105.0 ± 59.6^c^
Resistin (ng/mL)	2.75 ± 0.55^a^	5.71 ± 0.32^b^	3.09 ± 0.52^a^
IL6 (pg/mL)	0.095 ± 0.01^a^	0.270 ± 0.02^b^	0. 21 ± 0.016^c^
TNFα (pg/mL)	0.0089 ± 0.004^a^	0.04 ± 0.002^b^	0.012 ± 0.003^c^

Values represented as means and standard errors, the different superscript letters mean a significant difference at *P* > 0.05

**Fig. 1:**
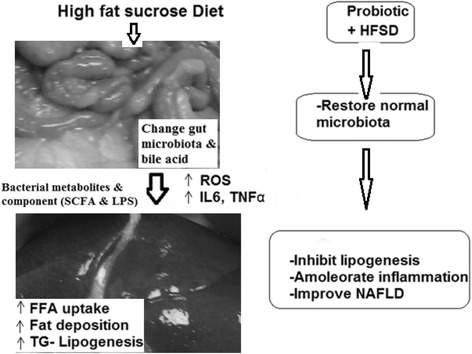
Mechanisms of HFSD-induced gut microbial alterations (via ROS, IL-6, TNF-α, SCFA, and LPS) and metabolic dysfunctions, and subsequent improvements mediated by probiotics

A photograph showing high fat deposition around the gut, and an enlarged liver, is presented in Fig. 1. This also shows the proposed mechanisms of HFSD-induced gut microbial alterations (via changes in levels of ROS, IL-6, TNF-α, SCFA, and LPS), as well as overall metabolic dysfunction and subsequent improvement via probiotic administration. This figure indicates that changes occurred in the gut microbiota and bile acid, due to the HFSD. These changes resulted in elevations in free fatty acid uptake and in the deposition of triglycerides, both of which induced NAFLD, as indicated by levels of ROS and inflammatory markers. Administration of probiotic restore the normal microbiota therefore, improve NAFLD via inhibition of lipogenesis and inflammatory markers.

**Fig. 2:**
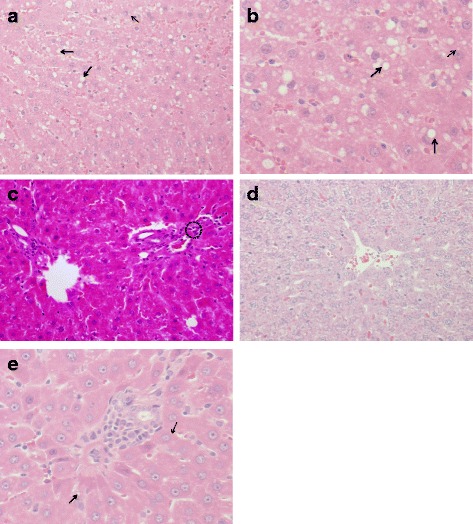
Histopathological results in the different groups of the experiment: **a** & **b** Microscopic analysis of the liver (H&E stain, magnification 20×) showed multiple fat globules between and within hepatocytes, with concomitant degenerative changes in hepatic cells within the HFSD group. Also NAFLD group revealed, macrovesicular steatosis (*bold line arrow*): major fat droplets are existing in hepatocytes; microvesicular steatosis (*dotted arrow*): minor fat droplets are present in hepatocytes. **c** Liver focal periportal inflammation (magnification 40×) and aggregation (*cluster*) of inflammatory cells (*within dotted circles*) in NAFLD group. **d** Typical histological structure of normal hepatic cells, with no inflammatory cells in the perivenular area, within the normal group. **e** View of hepatic cells with normal shape and lower fat globules in the HFSD and probiotic mixture groups

The hepatic histopathological studies showed multiple fat globules, mild to moderate macro and microvesicular steatosis, degenerative changes, and focal periportal inflammation in the hepatic cells of the HFSD group (Fig. 2a, b and c). By contrast, Fig. 2d reveals the normal histological structure (non detectable macro and microvesicular steatosis) that was typical of hepatic cells observed in the normal-diet group. Figure 2e illustrates the return of normal structure in the hepatic cells of the probiotic-receiving group.

Concerning, NAFLD score, normal group had 0 score for 90% of the cases and HFSD group revealed an increases in the number of cases in the score 1 followed by 2 (mild to moderate steatosis) while, probiotics group presented increases in the number of cases in score 0 and 1, that indicate improve in the steatosis score (Table 5).

**Table 5:** Histopathological features of normal, HFSD-fed rats and HFSD + Probiotic cases n (%)

Steatosis Score	Normal (*n* = 20)	HFSD (*n* = 20)	HFSD + Probiotic (*n* = 20)
0 (<5%)	18 (90%)	0 (0%)	11 (55%)
1 (5–33%)	1 (5%)	12 (60%)	7 (35%)
2 (>33–66%)	1 (5%)	7 (35%)	2 (10%)
3 (>66%)	0 (0%)	1 (5%)	0 (0%)

0, 1, 2 and 3 indicate, steatosis score, while 5, 5–33,33–66 and 66% represent percentage of the total area affected in the examined slides

## Discussion

A mixture of probiotic strains was expected to have a greater benefit on the health of the host than a single-strain probiotic, due to their presumed synergistic effects and complementary actions within the gut. In addition, host animals would presumably show diverse signs of improved health and amelioration of metabolic syndromes, in agreement with the findings of Yoo et al. [20]. Our data indicated that the HFSD induced elevated serum lipid profiles for TG, TC, and LDL, compared to the normal diet (Table 2). These results agree with the findings of Amin et al. [9]. The increased levels in the lipid profiles may have been responsible for, or associated with, the accumulation of fat droplets observed in the livers of HFSD-fed animals; this seemed to lead to the development of various degrees of NAFLD, along with macro, microvascular steatosis and focal periportal inflammation (Fig. 2a, b and c).

Diets such as the HFD can induce changes in the conformation of gut microbiota which can increase gut permeability, reduce bacterial LPS removal, increase levels of bacterial components, and induce metabolic endotoxemia. Furthermore, this endotoxemia may result in subclinical inflammation, leading to numerous metabolic dysfunctions [7, 13, 30, 31], which can further result in disturbances in fat metabolism. These disturbances can be indicated by increases in free fatty acid uptake, triglyceride lipogenesis, and fat deposition in the hepatic tissues. This condition may also lead to hepatosteatosis, with high serum lipid profiles and increased lipogenesis.

Administration of the probiotic formulation appeared to stabilize or reverse the increased values in lipid profiles, and stabilized or reversed the incidence of steatosis, that had been observed in the HFSD groups. These changes were associated with improvements in liver function, serum TG levels, and leptin levels that control fat metabolism during exposure to HFSD. Another reason for observed improvements was the regulation of the gut microbiota; in terms of quantity, a return to a normal state was observed following the disturbances that were noted as a result of exposure to the high fat diet [9, 21].

Generally, the mechanism of probiotics effects may be governed by three principles: 1) it benefits both the host’s innate and acquired immunities, 2) it can restore the gut microbiota equilibrium between pathogenic and commensal bacteria, and 3) it can reduce the levels of toxins generated from the either the host microbiota or the food component [32]. A proposed mechanism for HFSD-induced gut microbial alterations is via changes in levels of ROS, IL-6, TNF-α, SCFA, and LPS, and their associated metabolic dysfunctions and the improvements generated by probiotics [5, 10] (Fig. 1). The normalizing effects of probiotic administration on serum lipid profiles is through inhibition of lipogenesis. These findings were consistent with those of Wu et al. [33], who found that *L. plantarum* K21, as a probiotic, produced cholesterol-lowering and bile salt-hydrolyzing capacities.

Serum leptin levels were significantly increased in the HFSD group compared to the normal diet group, while probiotic administration ameliorated this change. Leptin is considered the major hormone in adipose tissues, and its high levels explain the extraordinary amount of fatty tissues observed in the livers and intestines of rats that were fed the HFSD. Higher leptinemia may result in insulin resistance, and may aggravate the conditions of NAFLD. HFSD-induced elevations in serum resistin were due to increased fat deposition in the hepatic tissues, while probiotic administration resulted in decreases in serum resistin levels. This may have been due to disruptions in hepatic fat deposition, and improvements in hepatic steatosis. Resistin level decreases within the probiotic-administered group may have played an important role in reversing the accumulation of hepatic fat; however, the molecular and biochemical basis of the relationship between probiotics and resistin levels has yet to be defined.

Probiotics facilitate the restoration of normal hormonal activity in adipose tissues.

AST and ALT activity levels are the best clinical biomarkers of hepatic functions and illnesses [34]. Rats fed the HFSD showed alterations in ALT activity levels when compared with rats fed the normal diet (Table 2); this was apparently a consequence of increased lipogenesis, increased accumulation of lipids in the hepatic cells, and increased focal periportal inflammation (Fig. 2a, b and c). Additionally, enzymatic transaminases from cells are liberated into the blood due to the damaging effects of the presence of fat droplets, and also due to the elevated amount of ROS generated from lipid peroxidation.

Our results were consistent with previous studies that had indicated that *R. verniciflua* administration significantly reduces ALT activity levels in carbon tetrachloride-treated livers, and may be effective in reducing liver inflammation [35]. Similarly, chronic alcohol consumption may produce gut dysbiosis and may enhance the permeability of the intestines to endotoxins such as alcohol-generated acetaldehyde, which can interrupt constricted junctions. These changes induce deteriorations in the intestinal microvilli, but retreatment of mice with probiotics normalized the intestinal microvilli and restored connections [36, 37].

The present work showed that the HFSD induced increases in markers of NAFLD, hypertriglyceridemia, and hyperglycemia, and also generated deleteriously elevated levels of liver function markers, final body weights, and body weight gains, while the administration of a probiotic mixture could counter these changes. Previous researchers showed the effects of different probiotics on the modulation of the gut bacterial structure and strain composition when facing disturbances resulting from a high dietary fat diet. HFD-induced metabolic syndrome is due to structural disruptions of the intestinal microbiota, combined with inflammatory factors [38, 39]. *Lactobacillus* and *Bifidobacterium*, as probiotics, diminished body weight gain and macrophage penetration into epididymal fatty tissues, clearly enhanced glucose-insulin homeostasis, and improved hepatic steatosis (Fig. 2e). These bacteria shifted the overall structure of the gut microbiota that had been disturbed by the HFD towards that of lean mice fed a normal diet [19]. This is consistent with the finding that, overall, microbes may directly influence fatty acid uptake and satiety responses either through regulation of enteroendocrine cell numbers, or through direct regulation of gut peptide hormone production and secretion [40]. Our data revealed that TNF-α and IL-6 levels in the serum of the HFSD group were significantly increased, compared to those of the group fed the normal diet (Table 4). However, when given the probiotic mixture, the levels of these cytokines in the HFSD group returned to normal.

Intestinal microorganisms affect cellular metabolism in different hepatic and adipose tissues outside the gut; in this way they can control the glucose and lipid homeostasis, and the general inflammatory status, of their host [17]. Ingestion of a high-fat diet produces modifications within the intestinal microbiota and increases gut-derived inflammatory agents by renewing bowel flora in conditions of high fat diet-induced steatosis. However, the progress of inflammation is also an important factor, as it is associated with the appearance of hyperphagia, obesity [38], and NAFLD, as a consequence of changes in diet and microbiota.

The main type of fatty acid used in the HFSD of this experiment was a long chain saturated palmitic acid. The nature of the diet ingested, especially with high-fat diets, can lead to endotoxemia, which results from increased bacterial LPS amounts in the blood [39]. Stimulation of fat cells with LPS, or with different kinds of fatty acids (palmitic, myristic, or linoleic acids), has shown that palmitic and stearic acids are capable of generating inflammation, either alone or synergistically with LPS; however, these fatty acids combined with LPS induce greater increases in IL-6 levels than does LPS alone [41]. Fatty acids, either short or long chain, saturated or unsaturated, can affect the stimulation of immunological cells in the intestine, which in turn can produce various inflammatory reaction patterns [42, 43]. For example, long-chain saturated fatty acids bind to toll-like receptor 4 (TLR-4) and induce pro-inflammatory cytokine expression in macrophages [44]. Increased production and secretion of cytokines such as TNF-α and IL-6 may be due to the incorporation of specific fatty acid types, such as palmitic saturated long chain fatty acid, into the cell membrane. This can alter the configuration of lipids within the lipid bilayer, resulting in alterations or displacements of biosignaling proteins from the lipid raft, and modifying the stimulation of such receptor proteins as TLR-4 [45]. Some fatty acids induce low grade inflammation [41]; therefore, although soft butter may positively disturb normal blood lipids (lipemia), the high amount of SFA may activate inflammation independently from LPS action. Indeed, the long chain saturated palmitic and stearic fatty acids that were used in the HFSD of this study are able to trigger the release of inflammatory biomarkers such as IL-6 and TNF-α.

Overgrowth, strain specificity, and/or low microbiota richness inside the small intestine may stimulate the internal formation of ethanol, lead to choline insufficiency, and increase bacterial LPS. These factors are involved in the process of intestinal barrier damage, which may enhance the regular interactions between gut microbiota and may increase the occurrence of some products and hepatic receptors, such as TLR. This process may result in stimulating a cascade of actions leading to NAFLD, resulting in inflammation and hepatic cirrhosis [46]. These effects are indicated by the elevation of lipid profiles and inflammatory markers in blood serum.

In summary, HFSD, fatty acid type, hyperleptinemia, microbiota overgrowth (and its consequent metabolites), and LPS, were able to enhance the production of the inflammatory biomarkers IL-6 and TNF-α; this induced and aggravated hepatosteatosis, thus allowing for progression toward different degrees of fibrosis in the experimental animals. These results agree with those of Hong et al. [47], who reported that probiotics down-regulated the alcohol-induced expression of TLR-4 in animals fed normal diets and HFD. Additionally, alcohol-induced liver diseases could be effectively treated using probiotics to regulate the gut-liver axis. Using a cocktail of probiotics, we found that *Bifidobacterium* and *Lactobacillus* strains affected host inflammation, adipose tissue hormone levels, and intestinal microbial composition. Treatment of HFSD-induced NAFLD with different probiotic mixtures induced modifications within the intestinal microbiota that attenuated metabolic disruptions by reducing serum lipid profiles and inflammatory biomarkers. Our work provides novel data suggesting that multistrain probiotics, composed of a mixture of *Lactobacillus plantarum*, *Bifidobacterium*, and *Bacillus subtilis* strains, would be effective in treating NAFLD.

## Conclusions

In conclusion, the probiotic formula used in this study was effective in the treatment of NAFLD by ameliorating increased lipid profiles, liver function markers, inflammatory markers, and leptin and resistin hormone levels. Probiotics constitute potential therapeutic and nutritional treatments that allow the control of hepatosteatosis and associated disorders. There are links between the gut-liver axis, fat metabolism, hormonal balances within adipose tissues, and inflammatory mediators that results in hepatic steatosis and associated disorders. Understanding these links, and the different mechanisms that are involved, requires further investigation. Future studies that search for the best probiotic strains, doses, and algorithms of administration are needed. Probiotic administration shows great potential as a treatment for hepatic diseases; this justifies more research in the future. Further studies in humans are also needed.

## Abbreviations

GSH: Glutathione
HDL: High density lipoprotein
HFSD: High fat-sucrose diet
IL6: Interleukin 6
LDL: Low density lipoprotein
LPO: Lipid peroxidation
NAFLD: Non-alcoholic fatty liver disease
ROS: Reactive oxygen species
SCFA: Short chain fatty acid
TLR4: Toll-like receptor 4
TNFα: Tumor necrosis factor alpha
